# An investigation of the use of app technology to support clinical management of patients with chronic myeloid leukaemia (CML)

**DOI:** 10.1177/10781552221090904

**Published:** 2022-04-11

**Authors:** Saffiya Khadam, Teresa Chu, Nigel Deekes, David FitzGerald, Andrea Preston, Nick Duncan

**Affiliations:** 1 4490 School of Pharmacy, University of Birmingham, Birmingham, UK; 2 1732 University Hospitals Birmingham NHS Foundation Trust, Birmingham, UK; 3 Patient representative; 4 University Hospitals Bristol and Weston NHS Foundation Trust, Bristol, UK

**Keywords:** Chronic myeloid leukaemia, patient education, app technology, eHealth

## Abstract

**Introduction:**

The availability of healthcare apps to support patient self-management of various medical conditions, including cancer, has increased considerably in the past decade. However, there are limited published data on the role of apps in the management of chronic myeloid leukaemia (CML). The primary aim of this study was to investigate the current and future role of apps as a means of supporting patients with CML.

**Methods:**

A 31-item questionnaire was developed and distributed to patients via three on-line CML support groups.

**Results:**

Responses were received from 286 patients. There was an approximate 2:1 female: male split and the majority (54%, n = 155) resided in the United Kingdom. 91% (n = 260) of respondents were currently receiving drug treatment for their CML. 23.4% (n = 67) of respondents were aware that apps were available to support their CML management and 11.5% (n = 33) had experience of using such an app. 94.1% (n = 238) of those who had not used a patient support app in the past stated that they would consider using an app in the future to help manage their disease. App awareness was significantly higher amongst male patients (30.3% vs. 19.9%). Likelihood of being a current or previous app user was higher amongst younger patients (16.3% for <55 years old vs. 5.6% for ≥55 years old) whilst younger patients and those with a more recent diagnosis of CML were both more likely to be interested in using an app in the future. When asked about potential app functionality, a drug interaction checker was the feature of greatest interest to respondents.

**Conclusions:**

We have identified both a lack of awareness of and a low uptake of patient support apps amongst CML patients. Importantly, we have demonstrated a clear interest in CML-specific apps amongst this population. Based on the functionality that study participants were most interested in, we will work with health care professionals, app developers and patients to develop a new app to deliver holistic support to CML patients.

## Introduction

In the past decade, there has been a significant increase in the availability of healthcare apps and other eHealth strategies to support patients with a variety of medical conditions, including cancer.^1,2^ Given the increasing ubiquitousness of smartphones and associated technological innovations, this is perhaps unsurprising, and reflects an increasing focus on providing patients with greater autonomy and responsibility for their health.^3^ Importantly, healthcare apps have the potential to empower patients to better manage their medical conditions and have been shown to offer concrete benefits such as increased knowledge, better symptom control and improved adherence rates.^4–8^ Within the healthcare app arena, there has been a particular focus on developing apps to support patients with chronic diseases such as diabetes and mental health conditions.^9^ Although cancer has not historically been considered as a chronic condition, recent improvements in drug treatments have meant that many patient groups may now require continuous treatment for many years (and potentially decades). This paradigm shift in management strategies has led to an increasing focus on key pharmaceutical care issues such as patient education^10^ and medication adherence^11^ as important drivers for delivering optimal patient outcomes in the cancer setting.

Chronic Myeloid Leukaemia (CML), is an example of a malignancy where the use of daily oral anticancer medicines, in the form of tyrosine kinase inhibitors (TKIs) targeting the BCR-ABL oncoprotein, have revolutionised disease management.^12,13^ As an illustration of this, patients can now be reassured at diagnosis that the vast majority will have a near normal life expectancy.^14^ However, there is compelling trial evidence that sub-optimal medicine adherence has a profound negative effect on clinical outcomes in CML^15,16^ and there is therefore a pressing need to ensure that these patents receive appropriate holistic support from specialist pharmacists and other members of the healthcare team.

Limited published data on the use of eHealth interventions (in the form of SMS medication reminders and on-line symptom recording) to support patients with CML, have demonstrated some benefits in relation to adherence and symptom management.^17,18^ Additionally, a large survey of CML patients showed that mobile phone reminders were a relatively highly ranked strategy as a potential means of improving adherence.^19^ However, whilst we are aware of two CML-specific mobile phone apps currently available to support patients (CML Life and CML Today), we are not aware of any data on the level of patient engagement with and interest in using such apps as these. Importantly, although there are published data on desirable app content amongst cancer patients in general,^20,21^ what functionality CML patients value in an app is also unknown. Consequently, we decided to undertake an international questionnaire survey in this area with the principal aims of our study being as follows: firstly, to investigate the awareness of, usage of and interest in CML-specific apps amongst patients and, secondly, to determine what functionality and features CML patients would like to be present in a new CML-specific app.

## Methods

To try to maximise the response rate in a time-efficient manner, we employed an on-line questionnaire-based methodology for this study. A 31 item questionnaire (see Appendix), designed for online completion, was prepared using the www.onlinesurveys.ac.uk survey tool. All study authors contributed to the design, content and pre-launch piloting of the questionnaire. Having two patients with a diagnosis of CML as part of the study team ensured a strong level of patient and public involvement (PPI).

The questionnaire was split into three sections. Section one covered patient demographics and section two contained CML-specific questions relating to time since diagnosis and current and previous drug treatment. The main body of the questionnaire (section three) contained questions on app awareness, current/previous app use and future interest in using an app to support CML management. It also asked respondents to provide their opinion on potential app features and functionalities. The majority of questions were closed questions but, where appropriate, space for free text responses was also built in.

A news item with a link to the on-line questionnaire was posted on three large CML patient support websites in November 2019 and the link was kept open for 12 days. When the study closed, the questionnaire data were transferred to SPSS 26.0 (April 2019). Data analysis was predominantly of a descriptive nature. Both ordinal and nominal data were described through frequencies and percentages. Pearson's chi-square test was used to examine the relationships between nominal variables with a p-value of ≤ 0.05 being considered statistically significant.

Patients with a diagnosis of CML were eligible to complete the questionnaire. To promote enrolment, there were no additional inclusion or exclusion criteria set. Background information to the study (as an alternative to a specific participant-information sheet) was provided on the introductory page of the on-line questionnaire. From a consent perspective, before starting the questionnaire, participants were required to acknowledge electronically that they were happy to proceed and for their responses to be used in the study based on the information that had been provided to them. In order to ensure anonymity of the questionnaire data, no patient identifiers were collected. Ethical approval for the study was granted by the University of Birmingham School of Pharmacy Ethics Committee (Ref: 2019-05).

## Results

### Respondent demographics

A total of 286 questionnaire responses were received. The majority (56%) of respondents were <55 years old and there was an approximate 2:1 female:male split. Just over half (54.2%) of respondents were from the United Kingdom, with North/Central America being the second most common geographical location (26.6%). Approximately two-thirds of respondents had been diagnosed with CML in the past 5 years and 90.6% were currently receiving drug treatment for their condition, with imatinib being the most commonly prescribed agent. Relevant demographic data are summarised in Table 1.

**Table 1.** Summary of respondent demographics.

	% of respondents (n = 286)
*Gender*	
Female	65.0% (n = 186)
Male	34.6% (n = 99)
Non-binary	0.3% (n = 1)
*Age group*	
<35	9.8% (n = 28)
35–54	46.2% (n = 132)
55–74	41.6% (n = 119)
75 and older	2.1% (n = 6)
Prefer not to say	0.3% (n = 1)
*Country of residence*	
United Kingdom	54.2% (n = 155)
North/Central America	26.6% (n = 76)
Rest of Europe	9.1% (n = 26)
Asia	4.5% (n = 13)
Australia	2.4% (n = 7)
South America	2.4% (n = 7)
Africa	0.7% (n = 2)
*Time since diagnosis*	
<1 year	17.5% (n = 50)
1–5 years	49.3% (n = 141)
6–10 years	16.8% (n = 48)
>10 years	16.4% (n = 47)
*Current drug treatment*	
Imatinib	35.7% (n = 102)
Nilotinib	23.1% (n = 66)
Dasatinib	19.6% (n = 56)
Bosutinib	9.1% (n = 26)
Ponatinib	1.7% (n = 5)
Other^a^	1.4% (n = 4)
Not on treatment	9.4% (n = 27)

^a^ ‘Other’ included Asciminib and hydroxycarbamide.

### App awareness and usage amongst CML patients

**Figure 1.**
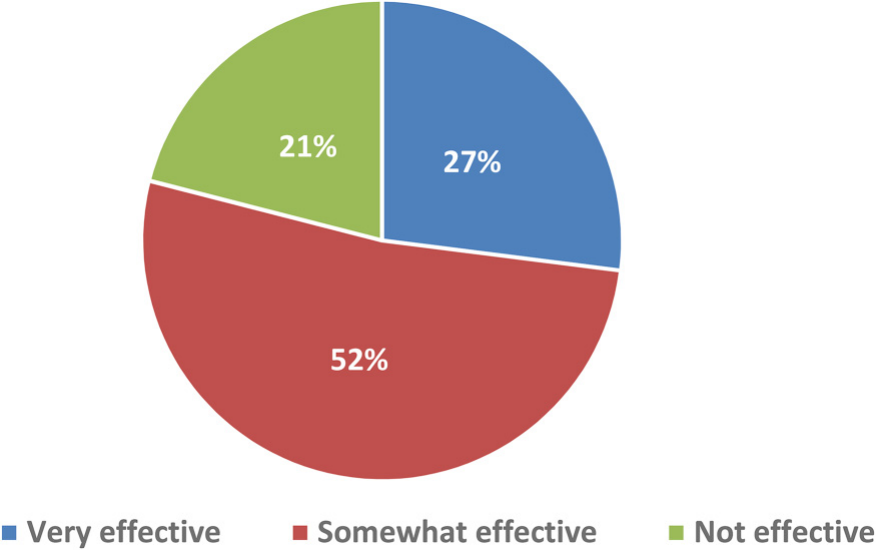
Opinion of apps amongst current and previous app users (n = 33) (color figure online).

In relation to app awareness, 23.4% of respondents (n = 67) stated that they were aware that there were apps available to support them in managing their CML. 11.5% (n = 33) of respondents were current (n = 22) or previous (n = 11) app users in this setting. Fifty eight percent (n = 19) of the current or previous app users had used one of the existing CML apps (CML Life or CML Today). The remaining users had either used a more generic app (e.g. Medisafe, EasyPill) or could not remember the name of the app. Data on the perceived effectiveness of the app(s) by current or previous app users are presented in Figure 1.

When app users (current and previous) were asked to name the feature they found the most useful, the top ranked features were reported to be ‘recording lab results’ (36%), ‘medication reminders’ (27%) and the provision of ‘links to useful articles’ (15%). A small proportion of respondents (6%) stated that none of the features were useful.

Of the 253 patients who had never used an app, 94.1% (n = 238) stated that they would consider using an app in the future to help them manage their CML. The remaining fifteen participants stated they were not interested in an app: the main reason for this was that they felt they did not need an app (n = 10). Other reasons included privacy concerns (n = 2) and a sense of unease about using apps (n = 3).

### Impact of demographics on app awareness, usage and interest

Table 2 presents data summarising the influence of age, gender and time since diagnosis on questionnaire responses. It can be seen that male participants were more likely to be aware of the existence of apps than female participants but age or time since diagnosis did not have an impact on app awareness.

**Table 2.** Impact of demographic factors on app awareness, usage and interest.

	Aware of apps	User of apps	Interested in apps
All patients	23.4% (n = 67)	11.5% (n = 33)	94.1% (n = 238)
Age <55 years	24.4% (n = 39)	**16.3% (n = 26)**	**97.0% (n = 130)**
Age ≥55 years	22.4% (n = 28)	**5.6% (n = 7)**	**90.7% (n = 107)**
Chi-square test	*P* = 0.696	***P* = 0.005**	***P* = 0.034**
Male	**30.3% (n = 30)**	13.1% (n = 13)	91.9% (n = 79)
Female	**19.9% (n = 37)**	10.8% (n = 20)	95.2% (n = 158)
Chi-square test	***P* = 0.048**	*P* = 0.550	*P* = 0.291
<6 years since diagnosis	21.5% (n = 41)	11.0% (n = 21)	**97.1% (n = 165)**
≥6 years since diagnosis	27.4% (n = 26)	12.6% (n = 12)	**88.0% (n = 73)**
Chi-square test	*P* = 0.656	*P* = 0.683	***P* = 0.008**

The sections in bold to indicate a statistically significant difference between groups.

In relation to app usage, younger participants (<55 years) were significantly more likely to be current or previous app users but the likelihood of being an app user was not influenced by gender or time since diagnosis. Participants with a relatively recent diagnosis of CML (<6 years) and younger participants (<55 years) were both more likely to be interested in using an app in the future but there was no significant difference between male and female participants in relation to this parameter.

### Prospective app features/functions

**Figure 2.**
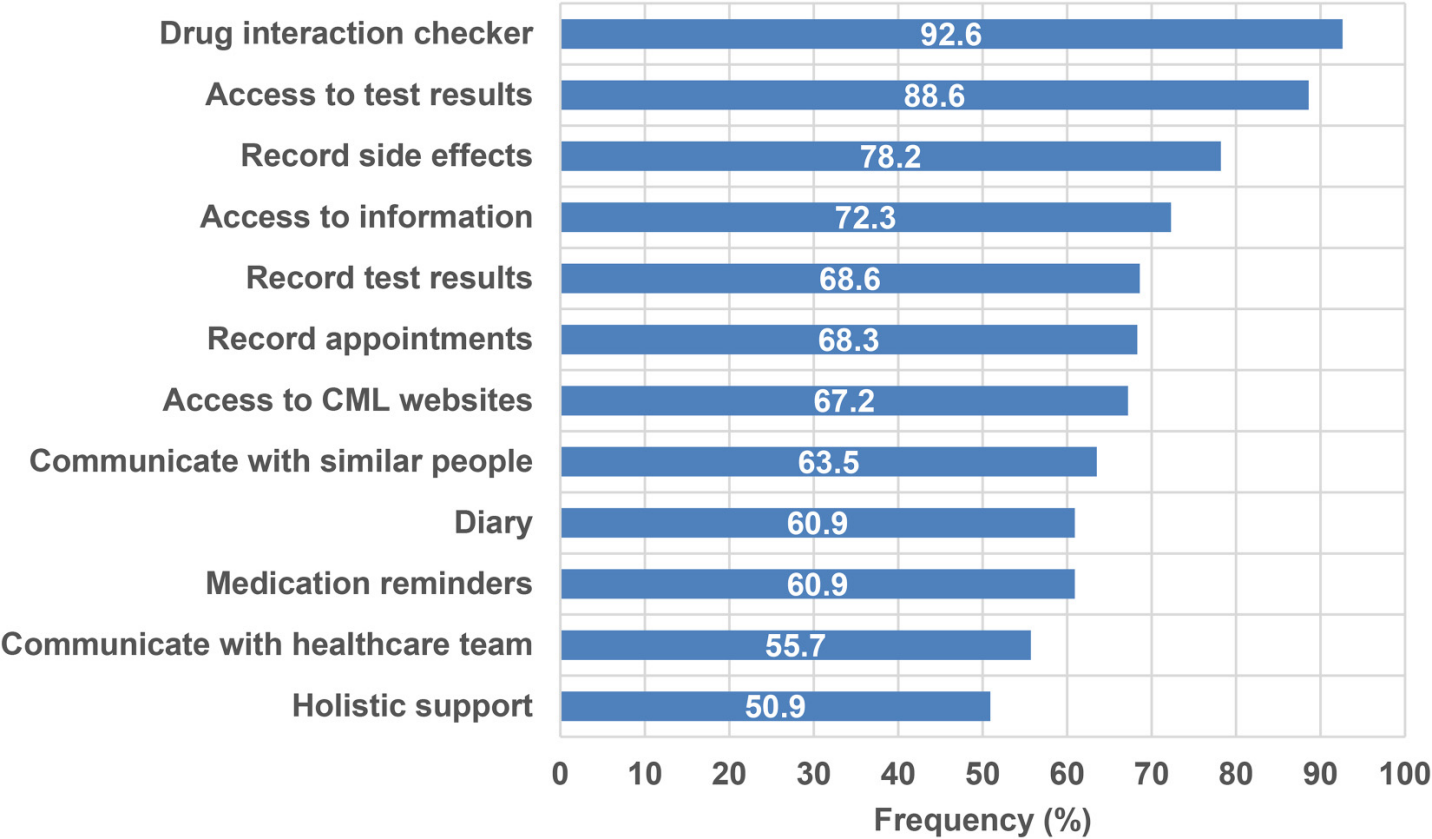
Percentage of respondents who were ‘very interested’ in app features, ranked according to popularity (n = 271)

With the exception of those who stated they were not interested in apps, all other participants (n = 271) were asked about potential content for a new CML-specific app. Thirteen potential features and functions were proposed in the questionnaire and respondents stated their level of interest in having these available on an app using the following categories: ‘very interested’, ‘somewhat interested’, ‘not interested’. These features/functions are ranked according to popularity in Figure 2. The most popular feature amongst participants was a drug interaction checker, with almost 93% stating that they were ‘very interested’ in this feature. All features were of interest to >50% of respondents.

### App recommendation and approval

When asked ‘what might encourage you to use an App to help you manage your CML?’ a recommendation from a consultant was found to be the most popular app encouragement strategy, being selected by 77% of respondents (see Table 3)

**Table 3.** Respondent opinion of different strategies to encourage app use.

	% of respondents (n = 271)
Consultant recommendation	77% (n = 210)
Patient recommendation	72% (n = 195)
Other Health care professional (HCP) recommendation	67% (n = 182)
Advert on charity website	60% (n = 162)
Advert on NHS website	34% (n = 92)

Finally, respondents were asked ‘How important is it to you to have the app approved by an organisation e.g. NHS Choices?’ The majority (54%, n = 147) stated that app approval by an organisation was ‘very important’, 36% (n = 96) felt that it was ‘somewhat important’, and the remaining 10% (n = 28) deemed it to be ‘not important’.

## Discussion

This large, international, questionnaire-based study has demonstrated relatively low levels of awareness of and use of health apps within the CML patient community. At the same time, it is apparent that interest in using apps within this patient group is very high, suggesting a significant unmet need in this area. Although CML-specific research in this area has not been undertaken previously, other studies have demonstrated relatively low levels of health app awareness amongst certain patient groups.^22,23^ Similarly, the finding that only 11.5% of respondents had used health apps, was not dissimilar to earlier studies investigating app use across various chronic conditions: app use in these studies was reported to be between 2.7%–29.2%.^24–26^ Additionally, this study showed that only about half of app users were current or previous users of either of the existing CML apps on the market. Reasons for this relative lack of interest were not specifically explored within the questionnaire, but it is noteworthy that neither of the apps in question offered any of the 3 most popular app functions as specified by the survey respondents (drug interaction checker, access to test results and ability to record side effects).

It was striking that 94.1% of respondents expressed an interest in using an app to help them manage their CML. Previous research investigating interest in apps has tended to focus on the mental health arena and has demonstrated interest levels in the range of 69–76%.^27–29^ The higher levels found in the current study may be partly explained by the more recent timeframe of the research, coinciding with greater availability of digital technologies in general. In fact, research has demonstrated that the COVID-19 pandemic led to a significant increase in interest in finding out about health apps amongst patients in the United Kingdom,^30^ and so it could be hypothesised that the level of interest in apps may in fact be higher still if the questionnaire was repeated at the present time.

This study revealed gender-related disparities in app awareness, with men being significantly more likely to be aware of apps than women (30.3% vs. 19.9%). However, this did not translate into a significant gender bias in relation to app usage or interest in apps. Interestingly, a previous study investigating mobile health technology knowledge and practices in an American population did not demonstrate a significant difference between male and female patients in app usage but did find that women were more aware of medical apps than men. Perhaps surprisingly, in light of previous research,^23^ age did not appear to be a factor in app awareness, with 24.4% of respondents under 55 years old reporting awareness of health apps compared to 22.4% of the older cohort. However, as has been demonstrated elsewhere,^23,27,31^ there was a marked difference (almost 3-fold higher) in app usage in favour of younger respondents and also a significant difference in relation to interest in using apps in the future. Consequently, any future developments in this area will need to take into account those factors that may underpin a reluctance to adopt digital health technologies amongst older patients.^32^

Finally, in relation to the influence of demographic factors on questionnaire responses, it was noteworthy that, although time since diagnosis did not appear to impact on app awareness or usage, patients who had been diagnosed in the past 5 years were much more interested in using an app in the future than those patients who had been diagnosed at least 6 years previously. Whilst accepting that the effect of age may have been a confounder in relation to interest in using an app, this supports the established principle that patients tend to need the greatest support shortly after diagnosis. It also ties in with the responses to an earlier question whereby the most common explanation for why respondents were not interested in an app was that they felt they did not need an app to support their CML journey.

With regards to potential functionality in a new CML app, we demonstrated that a drug interaction checker was the feature most desired by study respondents. All respondents were interested in having this built into the app with 92.6% stating that they were ‘very interested’ and the remaining 7.4% ‘somewhat interested’. Although previous research has also identified a drug interaction checker as a highly desirable app function,^33^ a large review of 424 health apps found that only 2.8% currently incorporated this feature.^34^ Given that all the common TKIs used to treat CML (imatinib, dasatinib, nilotinib, bosutinib and ponatinib) are metabolised by the cytochrome P450 system of liver enzymes, clinically significant drug interaction with these agents are a well-recognised concern^35–37^ and it is therefore particularly important that patients are equipped with tools to help them in this area. In addition, a number of respondents expressed interest in an interaction checker that could be utilised for substances other than drugs such as foods, vitamins and herbal remedies. There are a number of well recognised herbal and food interactions with TKIs, including interactions with citrus, pomegranate and black cohosh,^38^ and an interaction checker identifying more than just drug-drug interactions, could further aid self-management.

Survey respondents also expressed a high level of interest in being able to access and/or record test results on an app. CML is perhaps unique amongst cancers in that clinical response and disease burden can be accurately monitored by a relatively easily-measured molecular marker, the BCR-ABL transcript.^14^ Regular polymerase chain reaction (PCR) monitoring is mandated by current international guidelines^39,40^ as a means of allowing clinicians (and patients) to track progress against defined milestones of disease response. Although there may be technological and information governance challenges in providing a direct feed of blood results from a hospital computer system to a patient-help app, such approaches are becoming more common. However, even the ability for patients to manually record and track key results, such as their BCR-ABL transcripts, should be seen as desirable as a way of heightening self-awareness of their condition and supporting greater self-monitoring.^41,42^

In relation to the ability to record side effects, almost 80% of respondents felt this to be an important feature within an app. Furthermore, 60.9% of respondents were very interested in having a more generic diary feature that could be used to track symptoms, lifestyle changes and feelings. Previous studies have demonstrated a negative association between adverse effects and adherence to medicines in the setting of CML,^16,43,44^ and given the well documented link between suboptimal adherence and clinical outcome,^15,16^ it is important that early identification and monitoring of adverse effects is central to the overall patient monitoring strategy. Building symptom and side effect monitoring into digital health offerings has been shown to enhance side effect management in both CML and cancer more generally.^18,45^ Additionally, reporting side effects via an app may also allow for the collection of useful patient-reported outcome data, particularly in relation to less well documented adverse effects, where awareness amongst health care professionals may be relatively poor.^46^

Continuing the theme of strategies to improve adherence, just over 60% of respondents stated that they were very interested in having a medication reminder feature in an app. Although not as popular as some of the other features, this was still markedly higher than reported in a previous patient survey in which only 39% of CML patients stated that they used or would be interested in using a mobile phone medication reminder.^19^ The seven year time difference between this and the current study may help explain why the interest in a digital reminder feature had increased.

Almost three-quarters of respondents expressed a high level of interest in a feature that allowed access to background information on CML and its management, with the ability to access dedicated CML websites or on-line support groups also being ranked highly. The ability to communicate with other patients and, to a lesser extent, with healthcare professionals, were also of interest to respondents. Previous work has shown that health apps that provide access to disease-related information are highly valued by patients.^47^ Furthermore, the provision of such information can positively influence patient outcomes by increasing patient involvement and encouraging self-awareness and self-monitoring.^48,49^ Taken alongside the findings of a recent Taiwanese study that found that a lack of information concerning treatment was the main cause of poor medication adherence among CML patients,^50^ and the results of a large international CML patient survey that demonstrated a strong association between information provision and adherence,^43^ this supports the availability within an app of disease-specific information alongside appropriate signposting towards other potential channels of support. Providing interactivity in the form of in-app discussion forums or direct HCP links has been previously shown to be of interest to both patients and clinical teams^21,51–52^ but this may present obstacles in relation to data protection and patient confidentiality regulations and consideration must also be given to specific concerns on the part of patients about sharing information via an app.^20^

An appointment diary was another feature that was popular among participants. A recent systematic review investigated the effect of appointment reminders on healthcare outcomes and out of 56 included studies, 86% demonstrated appointment reminders to either increase attendance, increase cancellations well in advance or to decrease DNA (did not attend) rates.^53^ Consequently, it would be expected that such a feature would have a number of potential operational benefits in the CML setting also. Finally, and perhaps unexpectedly given that wellbeing and mindfulness are central tenets of one of the two existing CML patient apps, the level of interest in holistic support features (e.g. mindfulness podcasts) within an app was relatively low compared to the other proposed functionalities.

In relation to how best to encourage uptake of a CML-specific patient app, a personal recommendation from a HCP and approval by a specific organisation were seen to be of particular importance to study participants. Previous research has demonstrated high levels of acceptance of health apps amongst HCPs^54^ and there would appear to be merit in utilising them as a channel to encourage uptake of appropriate health apps by their patients. Furthermore, the recent increased focus on the formal assessment and validation of health apps by health care organisations would be expected to increase patient confidence in and uptake of such digital technologies.^55^

Strengths of this research study include its large size and international reach, both of which increase the generalisability of its findings. Building significant patient involvement into study design and promotion aided the recruitment process and ensured that the questionnaire content and presentation were both appropriate and understandable to the target audience. The primary limitation of the study was in relation to the use of an on-line questionnaire as the sole means of data collection as this could have excluded potential participants, either through a lack of access to technology or lack of awareness of the websites being used to promote the questionnaire. We are aware that just under a quarter of all new CML cases in the UK are diagnosed in people aged 75 and over^56^ and accept that our study population had relatively few patients within that age bracket. In future work, snowball sampling^57^ or the promotion of study instruments via health care professionals in a clinic environment may help overcome these limitations.

## Conclusions and future work

Through this large, international patient survey we have identified a lack of awareness of and a low uptake of patient support apps amongst patients with CML. Importantly, we have also demonstrated a clear interest in CML-specific apps amongst this population, as well as evidence in terms of a functionality ‘wish-list’ that the current available app technologies designed for CML patients do not appear to fully meet their needs. Because of this, and utilising our key findings, we are now working with specialist app developers to produce a new app for CML patients. The aim of the app (to be called ‘My CML’) will be to provide patients with a multi-purpose and user-friendly resource that empowers them to optimally manage their condition. This will be achieved by a suite of functionalities including a symptom/side effect tracker, a drug interaction checker, a medication reminder tool and an appointment diary, alongside the ability to record and generate graphs for key test results as well as links to key disease-specific and patient support information.

## Appendix: Questionnaire

An investigation of the use of App technology to support clinical management and adherence to medications in patients with Chronic Myeloid Leukaemia (CML)

I am a final year Pharmacy student at the University of Birmingham, and I am undertaking a project looking at the use of Apps in the CML setting. An App (application) is a programme that you can access on your mobile phone or online.

My supervisor and I have designed a questionnaire to help us understand your views on the use of healthcare Apps to support you in managing your CML. The answers you provide will form part of the preliminary research that may be used to develop an App that supports patients with CML. This questionnaire should take no longer then 5 to 10 min to complete.

### Disclaimer

Taking part in this research is voluntary. The answers you provide to questions will be anonymised and confidential.

### Consent

If you consent to taking part in this questionnaire and are happy to proceed please click on the link below and this will take you directly to the questionnaire.

This questionnaire is split into *THREE* sections:

Section 1: About You

Section 2: About your CML

Section 3: Your opinion on Apps

### Section 1: about you

1. What is your age group?
  □ 0–14
  □ 15–24
  □ 25–34
  □ 35–44
  □ 45–54
  □ 55–64
  □ 65–74
  □ 75–84
  □ 85 and over
  □ Prefer not to say
2. How would you describe your gender?
  □ Male
  □ Female
  □ Non-Binary
  □ Prefer to self-describe ……………………………
  □ Prefer not to say
3. Where do you live?
  □ United Kingdom
  □ Rest of Europe
  □ North America/Central America
  □ South America
  □ Africa
  □ Asia
  □ Australia
  □ Prefer not to say

### Section 2: your CML

1. How long has it been since you were diagnosed with CML?
  □ Less than 1 year
  □ 1–5 years
  □ 6–10 years
  □ More than 10 years
2. What drug treatment are you currently receiving for CML?
  □ Imatinib (Glivec/Gleevec)
  □ Nilotinib (Tasigna)
  □ Dasatinib (Sprycel)
  □ Bosutinib (Bosulif)
  □ Ponatinib (Iclusig)
  □ Other (please specify) ………………
  □ I don't know
  □ I’m not currently receiving any treatment
3. In the past have you received any other drug treatment for CML? Please select all that apply
  □ Imatinib (Glivec/Gleevec)
  □ Nilotinib (Tasigna)
  □ Dasatinib (Sprycel)
  □ Bosutinib (Bosulif)
  □ Ponatinib (Iclusig)
  □ Other (please specify) ………………
  □ I don't know
  □ I have not received any other drug treatment

### Section 3: your opinion on apps

1) Are you aware that there are Apps available to support you in managing your CML?
  □ Yes **Please continue to Question 2**
  □ No **Please continue to Question 8**
2) Do you currently use an App to help you manage your CML?
  □ Yes **Please continue to Question 2a**
  □ No **Please Continue to Question 3**
2a) Which App(s) are you currently using? Please select all that apply
  □ CML Life
  □ CML Today
  □ Other (please specify) …………………
3) Have you previously used an App to help you manage your CML?
  □ Yes. **Please continue to Question 4**
  □ No **Please continue to Question 8**
4) Which App(s) have you used in the past? Please select all that apply
  □ CML Life
  □ CML Today
  □ Other (please specify) …………………
  □ I can't remember the name of the App
5) How effective do you/did you find the App in helping you manage your CML?
  □ Very effective
  □ Somewhat effective
  □ Not effective
6) What features of the App(s) do you/did you use? Please select all that apply
  □ Medication reminders
  □ Recording if I’ve taken my medication
  □ Recording my lab results
  □ Symptom tracker
  □ Mindfulness podcasts
  □ Planner (to add and view appointments)
  □ Links to useful articles
  □ Other (please specify) ……….
7) Which feature of the App do you/did you find the **most** useful?
  □ Medication reminders
  □ Recording if I’ve taken my medication
  □ Recording my lab results
  □ Symptom tracker
  □ Mindfulness Podcasts
  □ Planner (To add and view appointments)
  □ Links to useful articles
  □ Other (please specify) ……….
  □ None of them were useful to me **Please continue to question 10**
8) Would you consider using an App to help you manage your CML?
  □ Yes **Please continue to Question 10**
  □ No **Please continue to Question 9**
9) What are the reasons behind this? Please select all that apply
  □ I have concerns surrounding my privacy/confidentiality
  □ I don't feel comfortable using Apps
  □ I don't have access to Apps on my phone
  □ I don't feel that I need an App
  □ I don't know
  □ Other (please specify) …………….. **End of questionnaire**

We are interested in developing a new App to support patients with CML. We would now like to ask you a few questions about the potential content of this App.

10) How interested are you in having a medication reminder feature on the App?
  □ Very interested
  □ Somewhat interested
  □ Not interested
11) How interested are you in accessing your hospital test results on the App e.g. BCR-ABL?
  □ Very interested
  □ Somewhat interested
  □ Not interested
12) How interested are you in recording your own tests results on the App e.g. BCR-ABL?
  □ Very interested
  □ Somewhat interested
  □ Not interested
13) How interested are you in having a diary feature on the App to track your symptoms/lifestyle/feelings?
  □ Very interested
  □ Somewhat Interested
  □ Not interested
14) How interested are you in recording your upcoming appointments on the App?
  □ Very interested
  □ Somewhat interested
  □ Not interested
15) How interested are you in the App having background information on CML and its’ management?
  □ Very interested
  □ Somewhat interested
  □ Not interested
16) How interested are you in having a feature on the App that allows you to access other kinds of holistic support e.g. mindfulness podcasts?
  □ Very interested
  □ Somewhat Interested
  □ Not interested
17) How interested are you in having a feature on the App that allows you to access specific dedicated CML websites/social media groups?
  □ Very interested
  □ Somewhat Interested
  □ Not interested
18) How interested are you in having a feature on the App allows you to communicate with other people in a similar situation e.g. in App discussion forums?
  □ Very interested
  □ Somewhat interested
  □ Not interested
19) How interested are you in being able to record medication side effects on the App?
  □ Very interested
  □ Somewhat Interested
  □ Not interested
20) How interested are you in the App having a feature that checks for drug interactions?
  □ Very interested
  □ Somewhat Interested
  □ Not interested
21) How important is it to you to be able to communicate with your healthcare team on the App?
  □ Very important
  □ Somewhat Important
  □ Not important
22) Is there any content or features that we have not asked you about that you would find useful in an App to help you manage your CML?
23) What might encourage you to use an App to help you manage your CML? Please select all that apply
  □ Recommendation from your consultant
  □ Recommendation from another healthcare professional e.g. pharmacist, specialist nurse
  □ Advert on the NHS website
  □ Advert on a charity website e.g. CML support groups, Macmillan
  □ Recommendation from another patient
  □ Other (please specify) …………………..
  □ None of the above
24) How important is it to you to have the App approved by an organisation e.g. NHS Choices?
  □ Very important
  □ Somewhat important
  □ Not important

Thank you for taking the time to complete this questionnaire.

Every response is valuable to us. Such research cannot exist without individuals who are willing to share their opinions and views.
